# Development of huge keloid at donor site and recurrent keloid at graft site after excision of pubic keloid followed by split-thickness skin graft: A case report

**DOI:** 10.1097/MD.0000000000039018

**Published:** 2024-08-02

**Authors:** Jun Yong Lee, Su Ram Kim, Gyeol Yoo, Sang Oon Baek

**Affiliations:** a Department of Plastic and Reconstructive Surgery, College of Medicine, The Catholic University of Korea, Seoul, Republic of Korea.

**Keywords:** donor, grafts, keloid, recurrence, scar

## Abstract

**Introduction:**

Keloids are the result of abnormal tissue scarring that occur after skin injuries leading to pain, psychological distress, and impaired quality of life. Despite the high recurrence rate after surgical treatment, excision is often inevitable for symptom control.

**Patient concerns:**

A 32-year-old female presented with a huge keloid on the pubic area accompanied by severe pain, pruritus, and infectious discharge. She also had multiple keloids on her chest and shoulders, indicating a strong predisposition to keloid formation.

**Interventions:**

While high potential for recurrence was anticipated, surgical excision was inevitable for symptom control. Complete keloid excision followed by split-thickness skin graft was performed.

**Diagnosis:**

Pathological report revealed keloid accompanied by ruptured epidermal inclusion cyst.

**Outcomes:**

Although postoperative care was highly recommended for prevention of keloid recurrence, the patient refused any additional management due to her financial difficulties. At postoperative 8 months, mild degree of keloid or hypertrophic scar at marginal area of the graft was observed, suggesting the potential sign of keloid recurrence. The patient voluntarily discontinued the outpatient follow-up for 2 years, and then returned with huge keloid not only at the graft site but also at the donor site.

**Conclusion:**

Keloid with inflamed epidermal inclusion cyst can cause severe pain where surgical excision is unavoidable, regardless of the high potential for recurrence. Additional postoperative care is necessary to prevent recurrence. Furthermore, attempts to minimize new keloid formation at the donor site after split-thickness skin graft, such as thin skin harvest or selecting the scalp as the donor site, should be considered.

## 1. Introduction

Keloid is the result of aberrant tissue scarring typically occurring in injured skin, and is caused by the overgrowth of granulation tissue or collagen type III during the healing process.^[1]^ It can cause pain, itching sensation, aesthetic discomfort and psychological stress. In a severe case, it significantly impairs the quality of one’s life. This scar is known for frequent recurrence. After surgical monotherapy of the keloid, the recurrence rate is reported by 50% to 80%.^[2,3]^ Regardless of its high recurrence rate, surgical treatment is inevitable in severe cases.

The authors present a case of huge, severe keloid at the pubic area where surgical intervention was unavoidable for symptom control. This case eventually resulted in huge keloid formations at the donor site, as well as a huge recurrent keloid at the graft site after split-thickness skin grafting (STSG). Efforts to prevent recurrence at the primary region and the formation of new keloids at the donor site will be discussed in this article. To our knowledge, occurrences of such huge keloids recurring at the primary site while simultaneously developing anew at the donor site are very rarely reported in the literature.

## 2. Case report

This case report was approved by the Institutional Review Board of The Catholic University of Korea, College of Medicine (Approval No. OC24ZISE0024), and a written informed consent was obtained from the patient.

**Figure 1.**
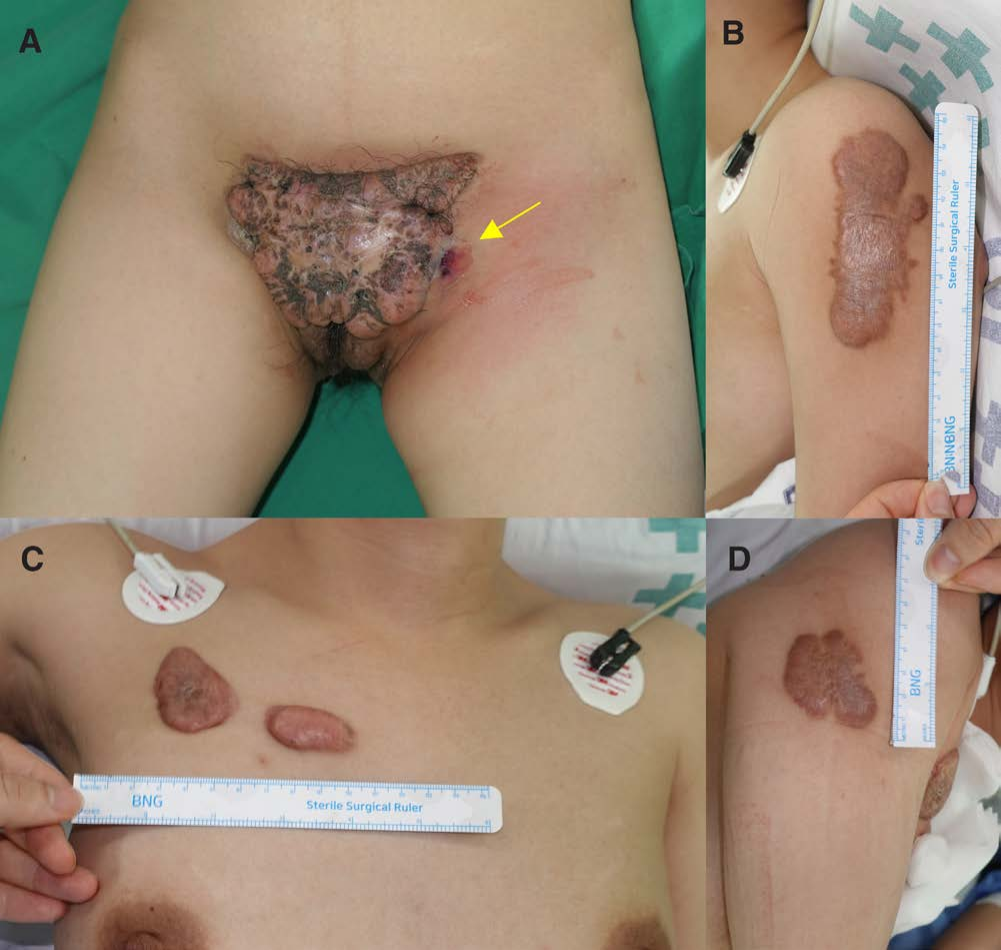
Preoperative clinical photograph. (A) Keloid observed in the pubic area. The dimension of the keloid was measured 15 × 8 cm^2^, accompanied by an infection sign on the left lateral border (indicated by yellow arrow). (B) Keloid observed in the left shoulder (8.5 × 2.5 cm^2^). (C) Keloids observed in the anterior chest (3 × 4 cm^2^, 3 × 2 cm^2^). (D) Keloid observed in the right shoulder (4 × 4 cm^2^).

A 32-year-old female suffered from a severe keloid on the pubic region for 10 years which gradually increased in its size (Fig. 1A). She also had other multiple keloids on chest and shoulders (Fig. 1B–D). It was obvious that her skin had a strong predisposition to keloid formation. The huge keloid on the pubic area was accompanied by severe pain, itching sensation, erythema, and infectious discharge. Surgical excision was inevitable due to severe pain and inflammation, regardless of the high potential for recurrence.

**Figure 2.**
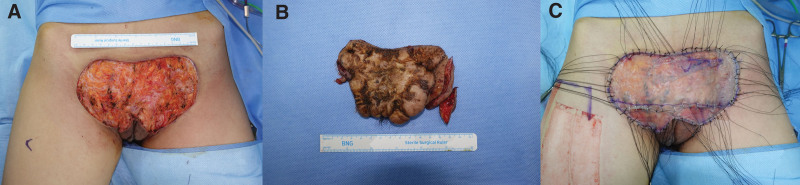
Intraoperative clinical photographs. (A) Defect size after keloid excision was measured 16 cm × 11 cm^2^. (B) Removed keloid. (C) Defect covered by one-stage Integra and split-thickness skin graft.

Total excision of the keloid resulted in a defect with the size of 16 × 11 cm^2^ which was covered by one-stage Integra (bovine, tendon collagen, and chondroitin-6-sulfate, INTEGRA Dermal Regeneration Template Single Layer, Integra LifeScience) followed by STSG with 0.2 mm thickness (Fig. 2). On fifth day postoperatively, skin graft was well-taken without complications.

**Figure 3.**
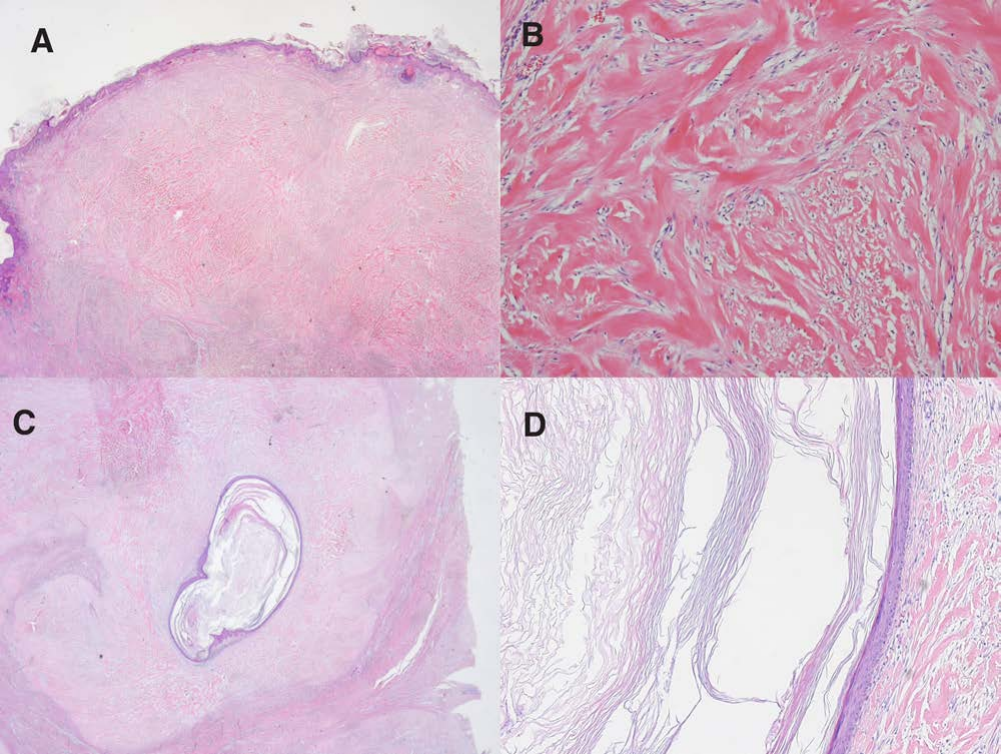
Histopathological findings. (A) Well-demarcated fibrous tissue involving the upper half of the dermis (H & E, ×10). (B) Nodules of thick, hyalinized keloidal collagen (H & E, ×100). (C) Ruptured epidermal inclusion cyst filled with keratotic material including keratin pearl (H & E, ×10). (D) ruptured epidermal inclusion cyst (H & E, ×200).

Pathological report revealed a well-demarcated fibrous tissue involving the dermis (Fig. 3A) with nodules of thick, hyalinized keloidal collagen (Fig. 3B) accompanied by a ruptured epidermal inclusion cyst (EIC) (Fig. 3C and D).

Immediately after surgery, the patient was recommended for postoperative silicone sheet application and radiotherapy for prevention of keloid recurrence. It was obvious that her skin was particularly prone to developing keloids based on her past history. However, due to her poor financial status, she refused any additional postoperative care. Her main issue was the inflammatory pain and pruritus rather than the keloid itself.

**Figure 4.**
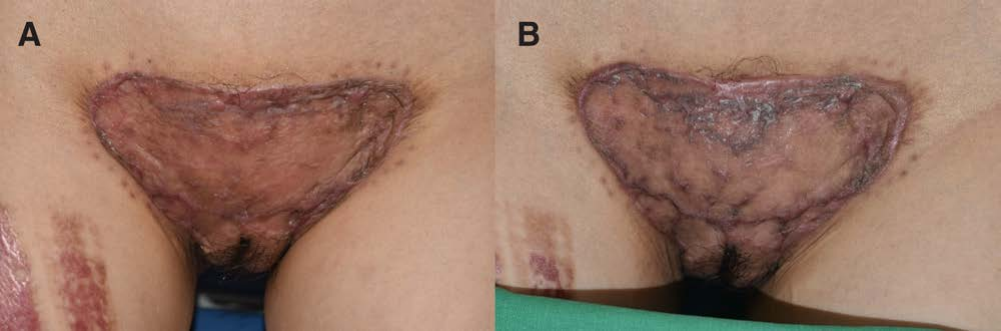
Postoperative clinical photographs. (A) At 2 months after the surgery, skin graft was stable without keloid recurrence. (B) At 4 months postoperatively, mild sign of keloid or hypertrophic scar was observed at the superior border of the skin graft.

**Figure 5.**
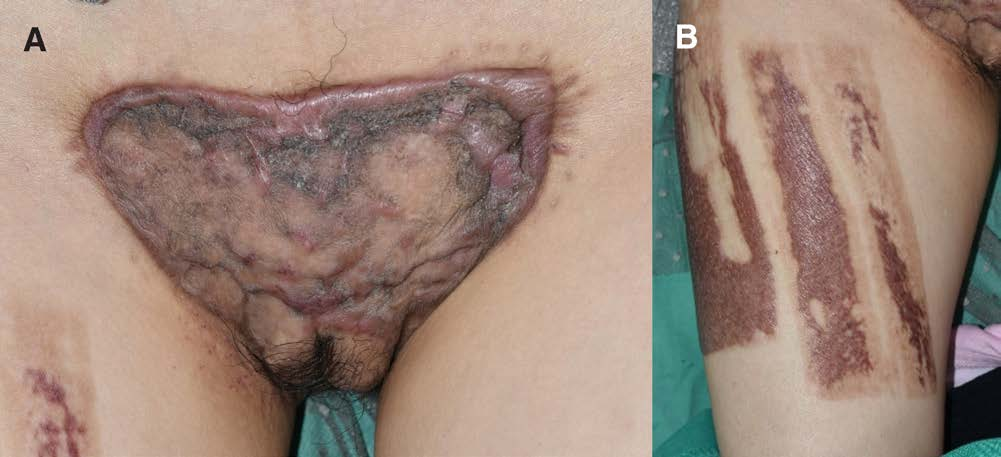
Clinical photographs at postoperative 8 months. (A) Increased size of protruding scar at the superior border of skin graft. (B) Mild protrusion of scar at the donor site of skin graft.

Two months after surgery, the skin graft was stable with no recurrence of keloid (Fig. 4A). At 4 months postoperatively, mild degree of keloid or hypertrophic scar was observed at the superior border of the skin graft (Fig. 4B). At postoperative 8 months, the protruding scar at the superior border of skin graft was increased in its size (Fig. 5A). Mild protrusion of broad scar at the donor site of skin graft in right thigh region was also observed (Fig. 5B). Despite the potential sign of keloid recurrence, the patient was still satisfied with the scar improvement and reduced pain. She voluntarily discontinued visiting the hospital after 8 months postoperatively.

**Figure 6.**
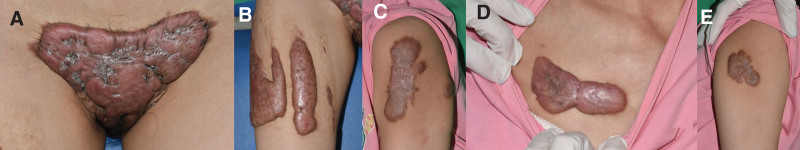
Clinical photographs at postoperative 2 years. (A) Huge recurrent keloid at the pubic region. (B) Huge keloid scar developed at the donor site of skin graft. (C) Keloid observed in the left shoulder. (D) Keloid observed in the anterior chest. (E) Keloid observed in the right shoulder.

At 2 years after the surgery, she returned to hospital with not only a huge recurrent keloid at pubic area (Fig. 6A) but also with the newly developed huge keloids at her right thigh which was the donor site of STSG (Fig. 6B). The keloids were again accompanied by pain and pruritus. The size of keloid scars on the chest and both shoulders had also increased when compared to 2 years ago (Fig. 6C–E).

## 3. Discussion

Currently, various types of treatment for keloid exist. Corticosteroids including triamcinolone acetonide have been widely used as injection or plaster ingredients. Radiotherapy, cryotherapy, and laser therapy are also well known for keloid treatment. Surgical excision is a traditional method of removing keloids. Various methods of wound closure after surgical removal of keloids can be used including linear closure, flap coverage, W-plasty, Z-plasty, and skin grafting.^[4]^ Harvest of skin graft can sometimes cause significant keloid or hypertrophic scar formation at the donor site. Thompson^[5]^ suggested that donor sites of skin graft have propensity for hypertrophic scars that are cosmetically unfavorable. Nevertheless, huge keloids at the donor site have rarely been reported in the literature.

Keloid is a pathological state that occurs with overgrowing tissue from skin injury. It causes pain, pruritus, and discomfort, which can significantly affect the quality of one’s life. The exact mechanism of keloid producing the pruritus and pain is controversial. The proliferative and inflammatory cells, cytokines, and growth factors of keloid have been implicated in the development of pruritus.^[3]^ There is also a study demonstrating that pruritus and pain in keloids are a result of physical pressure of the collagen deposition on nerve fibers causing an entrapment neuropathy.^[6]^ Yang et al^[7]^ referred to the recurrent pain as a result of epidermal cyst underneath the keloids. Epidermal inclusion cyst (EIC) is one of the most common subcutaneous tumors that could be frequently observed in clinics, which occurs anywhere in the body.^[8]^ Trauma that caused the keloid may stimulate epithelial proliferation and create the cysts.^[7]^ EIC may become enlarged, inflamed, and infected. Inflamed EICs typically show signs and symptoms such as pain and erythema.^[9]^

The area around the perineum is warm, humid, and has poor hygienic environment. Keloid in such condition can easily accompany cyst inflammation, leading to the additional scarring.^[7]^ As a result, keloid on pubic area has a high potential to be aggravated. Surgical intervention is unavoidable in cases with large keloid with severe symptoms. However, high potential for recurrence after surgical therapy exists. The keloid recurrence at the margins of the skin grafts is the main problem associated with skin grafting. Furthermore, potential development for new keloid at the donor site cannot be overlooked.

Surgeons have explored various methods to reduce keloid complications at the donor site of skin graft. Harvesting thin skin graft can contribute to reducing risks of keloids, since the depth of skin injury determines the extent of scarring.^[10]^ Converse and Robb-Smith suggested that the thinner the graft the faster the healing of the donor site, and that the quality of final donor appearance is proportional to the rapidity of healing.^[5]^ Thompson, and Converse and Robb-Smith have shown improved outcome of the donor site with the application of thin split skin grafts.^[11]^ Back grafting method, harvesting an additional graft adjacent to the initial donor site and meshed to cover both donor sites at once, has also been introduced as a method of donor site management.^[12]^ Bian et al^[13]^ introduced thin STSGs regrafting on split-thickness skin donor sites and reported a satisfactory clinical result.

The choice of the STSG donor site is also an important factor in determining the final quality of donor scar. The donor site can be chosen from various areas, especially the anterolateral thighs, as well as the back, trunk, lateral arm, and even the scalp. The thigh, which is the most commonly chosen donor site, provides a large surface area and is easy to apply a mechanical dermatome due to its firm surface which the dermatome operator can push against.^[14]^ The scalp is known as a donor site where keloid formation is less likely.^[15]^ The use of the scalp as a donor site for skin grafts has been discussed for a long time, since the first discovery of STSGs.^[16]^ The rarity of keloid formation on the scalp is explained by the rich subcutaneous blood flow, which has the potential to prevent the formation of keloids and hypertrophic scars.^[15]^ On the other hand, there are several drawbacks to take grafts from the scalp. It is technically more difficult to harvest when compared to the thigh donor.^[16]^ Shaving the scalp before surgery is unavoidable, and there is a low risk of permanent alopecia (0.7%) as a complication.^[17,18]^ Using the scalp as a donor site in young female patients requires sufficient discussion with the patient prior to the procedure.

Long et al^[19]^ recommended postoperative radiotherapy at the early stage to prevent the recurrence at the margin area of the skin graft. Although our patient was not able to receive adjuvant treatment due to her financial difficulties, adjuvant therapy such as steroid tape application, steroid injection, and postoperative radiation should be considered to control the keloid recurrence.^[20]^ Pachuau et al^[21]^ reported favorable outcomes with no keloid recurrence nor development of donor site keloids in 55 patients with chest “lock” keloids who were treated by keloid debulking and STSG followed by postoperative radiotherapy at donor and recipient sites. In patients with keloid-prone skin, postoperative therapy after surgical excision is crucial to control the recurrence. Patients should be provided by full explanation regarding the potential for recurrence and the necessity of postoperative therapy before starting any surgical intervention.

Although postoperative care was not available for financial issue in our case, the exacerbation of keloids despite attempted surgical treatment sheds light on the complexities of keloid management, especially in patients predisposed to keloid formation. While performing STSG for keloid treatment in such patients, surgeons should focus on efforts regarding the surgical method and postoperative care to prevent recurrence at the primary site and the formation of new keloids at the donor site.

## Author contributions

**Conceptualization:** Sang Oon Baek.

**Data curation:** Sang Oon Baek.

**Formal analysis:** Su Ram Kim.

**Funding acquisition:** Sang Oon Baek.

**Investigation:** Su Ram Kim, Gyeol Yoo.

**Methodology:** Jun Yong Lee, Sang Oon Baek.

**Project administration:** Sang Oon Baek.

**Resources:** Sang Oon Baek.

**Software:** Jun Yong Lee, Su Ram Kim.

**Supervision:** Sang Oon Baek.

**Validation:** Gyeol Yoo, Sang Oon Baek.

**Visualization:** Jun Yong Lee, Su Ram Kim.

**Writing – original draft:** Su Ram Kim.

**Writing – review & editing:** Jun Yong Lee, Sang Oon Baek.
